# Understanding the Connection between Epigenetic DNA Methylation and Nucleosome Positioning from Computer Simulations

**DOI:** 10.1371/journal.pcbi.1003354

**Published:** 2013-11-21

**Authors:** Guillem Portella, Federica Battistini, Modesto Orozco

**Affiliations:** 1 Institute for Research in Biomedicine (IRB Barcelona), Barcelona, Spain; 2 Joint IRB-BSC Program in Computational Biology, Barcelona, Spain; 3 Departament de Bioquímica i Biologia Molecular, Universitat de Barcelona, Barcelona, Spain; University of Houston, United States of America

## Abstract

Cytosine methylation is one of the most important epigenetic marks that regulate the process of gene expression. Here, we have examined the effect of epigenetic DNA methylation on nucleosomal stability using molecular dynamics simulations and elastic deformation models. We found that methylation of CpG steps destabilizes nucleosomes, especially when these are placed in sites where the DNA minor groove faces the histone core. The larger stiffness of methylated CpG steps is a crucial factor behind the decrease in nucleosome stability. Methylation changes the positioning and phasing of the nucleosomal DNA, altering the accessibility of DNA to regulatory proteins, and accordingly gene functionality. Our theoretical calculations highlight a simple physical-based explanation on the foundations of epigenetic signaling.

## Author Summary

In Eukaryotic cells, control of the patterns of DNA cytosine methylation – a mechanism that acts on top of the genetic code – plays a key role in the regulation of gene expression. The large prevalence of DNA methylation in vivo, suggests a connection between the physical properties of methylated and un-methylated DNA with the control of gene expression. In this work we investigate the physical implications of DNA methylation in nucleosomal DNA, in particular its preferred location with respect to the nucleosome core-particle and the consequences of DNA methylation for the accessibility of the genetic material. We find that methylated DNA is less prone to form nucleosomes due to a reduced elasticity, especially when all methyl groups are pointing outwards from the nucleosome core, and that multiple methylation could give rise to changes in nucleosome positioning.

## Introduction

In eukaryotic cells gene function is modulated by a myriad of epigenetic marks and interactions with signal molecules that control synergistically the production of RNA and proteins. Epigenetic marks are a set of heritable but reversible chemical changes of the DNA and histones that can trigger gene silencing and activation. One of the most important epigenetic marks is DNA cytosine methylation, which occurs in 60–90% of the CpG content in mammalian DNA. In fact, most (CpG)s, except those in regions with large tracts of CpG steps (“CpG-islands”), are methylated [1], [2], and changes in the methylation pattern of DNA is a fingerprint of different pathologies, including cancer [3]–[10]. DNA methylation has a known role in gene expression regulation [11]–[13], but despite extensive work [14]–[24] its mechanism of action is not well understood [25], i.e. it is not clear whether and how the chemical properties of ^Me^C impact gene expression regulation. A popular explanation suggests that it regulates the action of proteins containing methylated-DNA binding domains [7]. However, the prevalence of DNA methylation and the magnitude of the changes in the methylation pattern occurring under pathological conditions points towards a more general mechanism [26], [27].

A promising hypothesis to rationalize the biological impact of cytosine methylation is that it affects the accessibility of the DNA within chromatin by modulating intrinsic nucleosome positioning. Although several works suggest that methylated DNA increases nucleosome rigidity [28]–[30] and that it is less prone to wrap around nucleosomes than normal DNA [30]–[32], recent genome-scale studies suggest that nucleosome-bound sequences are slightly enriched in methylated cytosines (^Me^C), which are placed in a subtle 10-base periodicity pattern [33]. It is thus unclear whether methylation intrinsically favours or disfavours nucleosome formation, whether it leads or not to changes in nucleosome positioning or phasing, and what is the preferential location (if any) of ^Me^C.

To shed light on these questions, we have performed a theoretical analysis of the impact of CpG methylation on the structure and stability of the nucleosome. We find that methylation of CpG steps decreases the stability of the nucleosome. Such effect increases with the number of ^Me^Cs, depends on the position of the ^Me^C with respect to the histone core, and can be explained from variations in the mechanical properties of methylated versus un-methylated DNAs. Our results reveal that methylation is sufficient to induce changes in phasing and/or positioning of the DNA around the nucleosome, which in turn might modify the accessibility of DNA sequences to proteins controlling gene expression. Our study helps understand the important role of methylation in gene expression regulation.

## Methods

**Figure 1:**
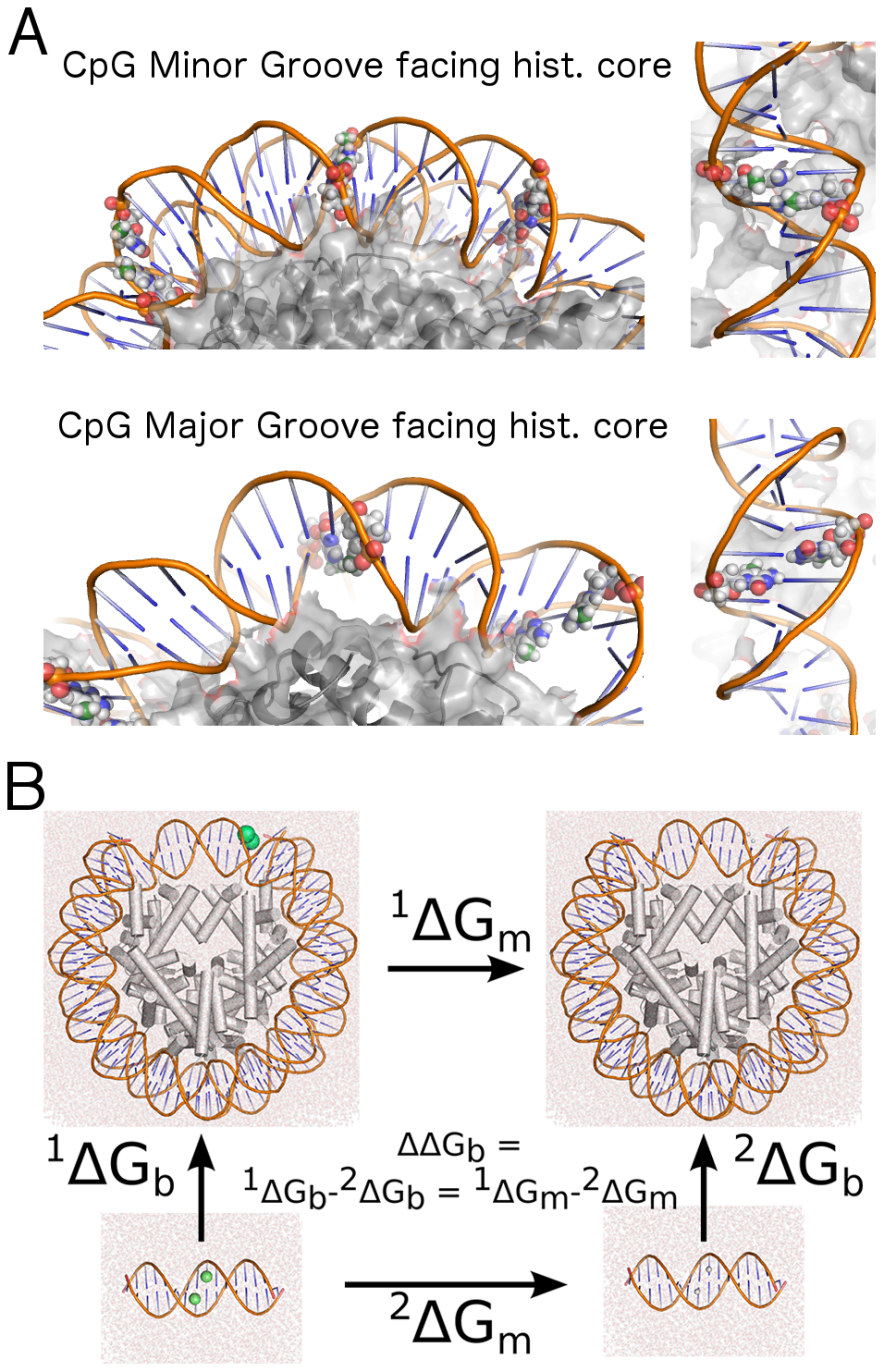
Mutation sites and thermodynamic cycle. (A) Example of two extreme cases of ^Me^C positioning (spheres) along the nucleosomal DNA with respect to the histone core (in grey) used in this work: (top) the CpG step minor groove faces the histones; (bottom) the CpG major groove faces the histones. The methyl carbon is colored in green. The images on the right show a lateral view of the DNA and the protein as seen from the solvent. (B) Diagram of the thermodynamic cycle used to extract the free energy variation (ΔΔG_b_ (kJ/mol)) in nucleosome-DNA stability due to methylation of CpG steps. The calculations of the unbound reference state for the single mutations were performed on shorter DNA chains, using the nearest 3 neighbors of the CpG steps in the nucleosome sequence, and 4 bases to cap the duplex termini (5′-CGAT and TACG-3′). As the histone proteins are not affected by the cytosine methylation in the unbound state, they were not included in the calculations related to such state. In case of multiple methylations, large fragments of different length were used (further details in SI material). The free energy difference associated with the removal of the methyl group is calculated using discrete thermodynamic integration (DTI). The methyl group is shown as a green sphere.

Molecular dynamics simulations and free energy calculations of fully solvated and neutralized mono-nucleosomes were carried on the X-ray structure with PDB code 1KX5 [34]. To save computational cost we have removed the long histone tails protruding out from the core. We subjected the energy-minimized structure to 200 ns of MD simulation, and we used the last structure to introduce different number of CpG and methylated CpG steps in positions described in Table S1 in Text S1. After energy minimization and initial thermalization, we performed MD for 100 ns for the selected single mutations and 200 ns for the multiple mutations (see table 1 and the next section), gathering information concerning solvent interaction or solvent densities, energies of stacking and geometrical parameters. Differential binding free energies were computed using the thermodynamic integration method in its discrete formalism, exploiting a thermodynamic cycle sketched in figure 1B. In this method, the free energy between two states is computed by integration of the derivative of the energy of the system as function of the state parameter λ, known as coupling parameter [35], which in our case describes either the methylated (e.g. λ = 0) or the unmethylated state (λ = 1). For each window we collected 9 estimates for 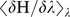 by using 9 blocks of 100 ps, which were then integrated through the entire mutation pathway to obtain mutation free energies (with associated statistical errors).

**Table 1:** List of the molecular dynamics simulations performed in this work.

Mutation type	Initial equilibration + SGTI	Discrete Thermodynamic Integration	MD simulations Nucleosome (un-methylated and methylated)	MD simulations unbound state oligo fragments (un-methylated and methylated)
Single mutation 1–10	10 ns equilibration + 10 ns SGTI	21 windows of 1 ns for each mutation, also for the reference state.	100 ns each	50 ns each
Single mutation 11–18	10 ns equilibration + 10 ns SGTI	21 windows of 1 ns for each mutation, also for the reference state.		
Multiple mutations	20 ns equilibration + 10 ns SGTI	21 windows of 1 ns for each mutation, also for the reference state.	200 ns each (except Mixed2)	50 ns each (except Mixed2)

For each mutation type, columns two and three describe the MD simulations that were performed to extract free energy differences. SGTI refers to slow growth thermodynamic integration method, see SI material methods. In columns four and five we list all the MD simulations that were carried out to characterize the effect of such mutation in terms of structural and energetic variations.

We measured the deformation energy for the methylated and un-methylated sequences using a mesoscopic energy model. This model describes the deformability along DNA helical parameters by an harmonic approximation, using the stiffness constants (*k_i_*) associated with the displacements with respect to the equilibrium values of the helical parameter [36], [37]. The values for the parameters describing the equilibrium geometry and stiffness constants of naked DNA were derived from long atomistic MD simulations (>200 ns, as found in the ABC consortium database [38]) of a reduced number of short DNA duplexes in water. The parameters for methylated cytosine were extracted from Perez *et al.* [31]. Full details on all computational methods and on the analysis performed are provided as SI text.

## Results/Discussion

### Free energy calculations indicate that nucleosomal DNA methylation disfavors nucleosome formation

We first studied the change in the stability of a nucleosome particle (histone proteins and DNA) induced by replacing cytosines with 5-methylcytosines in CpG steps located at representative positions along the DNA (examples in Figure 1A, full list in Table S1 in Text S1) by means of the thermodynamic cycle shown in figure 1B. The starting conformations for our free energy calculations were obtained from a 200 ns molecular dynamics (MD) simulation of the nucleosome in physiological conditions, using as initial conformation the highest-resolution X-ray structure available of the nucleosome [39].

We produced 18 different mutated nucleosome models, where each mutation consisted on placing a single CpG step at different locations where either the minor, or the major grooves face the histones (Figure 1A); these two types of positions explore widely different geometrical placements for ^Me^C in the nucleosome [40]. In addition, to study the effect of multiple methylations on nucleosomal stability we introduced several CpG steps simultaneously (see SI and Table S1 in Text S1). All the systems were extensively re-equilibrated prior to production runs. Nucleosomal and corresponding naked DNAs were used as starting points for TI calculations, where the reversible work associated with the methylation of the CpG step in nucleosomal and naked DNA was computed and processed to determine the change in nucleosome stability induced by cytosine methylation (see Figure 1B and Suppl. Information for details on all calculations performed in Text S1).

**Figure 2:**
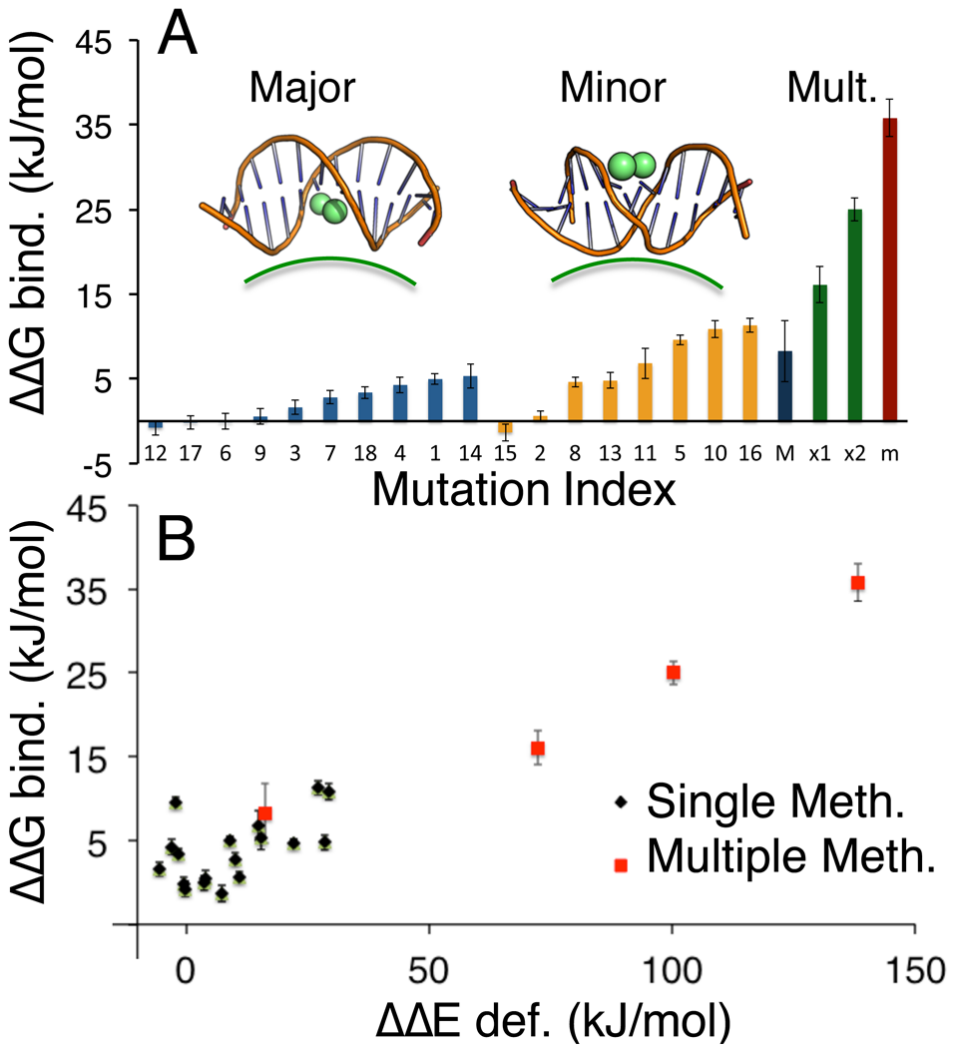
Differential binding free energy (ΔΔG bind. (kJ/mol)) of nucleosomal DNA. (A) Differential binding free energy values for single and multiple methylated CpG steps with respect to un-methylated sequences; methylations at major groove positions (blue) are better tolerated than at minor groove positions (light orange). Multiple methylations show a cumulative effect on the differential binding energy, following the trend of single methylations (methylation in major groove in dark blue, mixed major-minor groove in green, and minor groove in dark red). The exact location of each mutation is listed in Table S1 in Text S1. (B) Correlation between the variation in free energy (ΔΔG (kJ/mol)) and the variation in elastic energy (ΔΔE (kJ/mol)) for single (black dots) or multiple (red squares) methylated CpG steps in the nucleosomal DNA.

MD/TI calculations yield a positive free energy variation in all cases, demonstrating that methylation of DNA decreases the stability of the nucleosome (Figure 2), in contradiction with recent genome-wide-association study (GWAS) [33], but in agreement with many previous biophysical studies [25], [30], [32], [41], [42]. The disagreement with the GWAS conclusions could be attributed to an uncertainty of up to four base pairs in MNase-degradation nucleosome footprinting, which is close to half a DNA helical turn, and to the cell-to-cell variability of nucleosome positioning and methylations maps [43]. The ^Me^C-mediated destabilization of nucleosome is cumulative for multiple methylations, and in some cases the expected destabilization is so large (more than 20 kJ/mol) that it could challenge the entire stability of the nucleosome. Our MD/TI simulations also show that the effect of methylation on nucleosome stability is phase/position-dependent (Figure 2). In general, major groove methylations (i.e. those of CpG steps that face the histones through the major groove) are much better tolerated than minor groove methylations (i.e. those of CpG steps that face the histones through the minor groove). These results indicate that nucleosomes are more stable when the methyl groups in ^Me^CpG steps are placed pointing towards the histones and not to the solvent.

Analysis of the large amount of MD/TI data presented here (Figure 2) shows that methylation is especially nucleosome-destabilizing at some specific positions, such as those located at ±26 base steps from the nucleosome dyad position (mutations 10 and 16), where the nucleosome-bound DNA is characterized by a kinked geometry and a value of the roll angle (∼−7 deg. Fig. S1A in Text S1) that is widely different to the equilibrium value of ^Me^CpG steps (∼+14 deg.) [31], [44]. In comparison, methylation has a significantly lower stability cost when happening at major groove positions, such as −11 and 21 base pair from dyad (mutations 9 and 12), where the roll of the nucleosome bound conformation (+10 deg.) is more compatible with the equilibrium geometry of ^Me^CpG steps.

The nucleosome destabilizing effect of cytosine methylation increases with the number of methylated cytosines, following the same position dependence as the single methylations. The multiple-methylation case reveals that each major groove methylation destabilizes the nucleosome by around 1 kJ/mol (close to the average estimate of 2 kJ/mol obtained for from individual methylation studies), while each minor groove methylation destabilizes it by up to 5 kJ/mol (average free energy as single mutation is around 6 kJ/mol). This energetic position-dependence is the reverse of what was observed in a recent FRET/SAXS study [30]. The differences can be attributed to the use of different ionic conditions and different sequences: a modified Widom-601 sequence of 157 bp, which already contains multiple CpG steps in mixed orientations, and which could assume different positioning due to the introduction of new CpG steps and by effect of the methylation.

The analysis of our trajectories reveals a larger root mean square deviation (RMSD) and fluctuation (RMSF; see Figures S2–S3 in Text S1) for the methylated nucleosomes, but failed to detect any systematic change in DNA geometry or in intermolecular DNA-histone energy related to methylation (Fig. S1B, S1C, S4–S6 in Text S1). The hydrophobic effect should favor orientation of the methyl group out from the solvent but this effect alone is not likely to justify the positional dependent stability changes in Figure 2, as the differential solvation of the methyl groups in the bound and unbound states is only in the order of a fraction of a water molecule (Figure S5 in Text S1). We find however, a reasonable correlation between methylation-induced changes in hydrogen bond and stacking interactions of the bases and the change in nucleosome stability (see Figure S6 in Text S1). This finding suggests that methylation-induced nucleosome destabilization is related to the poorer ability of methylated DNA to fit into the required conformation for DNA in a nucleosome.

### Changes in the elastic deformation energy between methylated and un-methylated DNA correlate with nucleosomal differential binding free energies

To further analyze the idea that methylation-induced nucleosome destabilization is connected to a worse fit of methylated DNA into the required nucleosome-bound conformation, we computed the elastic energy of the nucleosomal DNA using a harmonic deformation method [36], [37], [44]. This method provides a rough estimate of the energy required to deform a DNA fiber to adopt the super helical conformation in the nucleosome (full details in Suppl. Information Text S1). As shown in Figure 2, there is an evident correlation between the increase that methylation produces in the elastic deformation energy (ΔΔE def.) and the free energy variation (ΔΔG bind.) computed from MD/TI calculations. Clearly, methylation increases the stiffness of the CpG step [31], raising the energy cost required to wrap DNA around the histone octamers. This extra energy cost will be smaller in regions of high positive roll (naked DNA ^Me^CpG steps have a higher roll than CpG steps [31]) than in regions of high negative roll. Thus, simple elastic considerations explain why methylation is better tolerated when the DNA faces the histones through the major groove (where positive roll is required) that when it faces histones through the minor groove (where negative roll is required).

### Nucleosome methylation can give rise to nucleosome repositioning

We have established that methylation affects the wrapping of DNA in nucleosomes, but how does this translate into chromatin structure? As noted above, accumulation of minor groove methylations strongly destabilizes the nucleosome, and could trigger nucleosome unfolding, or notable changes in positioning or phasing of DNA around the histone core. While accumulation of methylations might be well tolerated if placed in favorable positions, accumulation in unfavorable positions would destabilize the nucleosome, which might trigger changes in chromatin structure. Chromatin could in fact react in two different ways in response to significant levels of methylation in unfavorable positions: i) the DNA could either detach from the histone core, leading to nucleosome eviction or nucleosome repositioning, or ii) the DNA could rotate around the histone core, changing its phase to place ^Me^CpG steps in favorable positions. Both effects are anticipated to alter DNA accessibility and impact gene expression regulation. The sub-microsecond time scale of our MD trajectories of methylated DNAs bound to nucleosomes is not large enough to capture these effects, but clear trends are visible in cases of multiple mutations occurring in unfavorable positions, where un-methylated and methylated DNA sequences are out of phase by around 28 degrees (Figure S7 in Text S1). Due to this repositioning, large or small, DNA could move and the nucleosome structure could assume a more compact and distorted conformation, as detected by Lee and Lee [29], or a slightly open conformation as found in Jimenez-Useche et *al*. [30].

**Figure 3:**
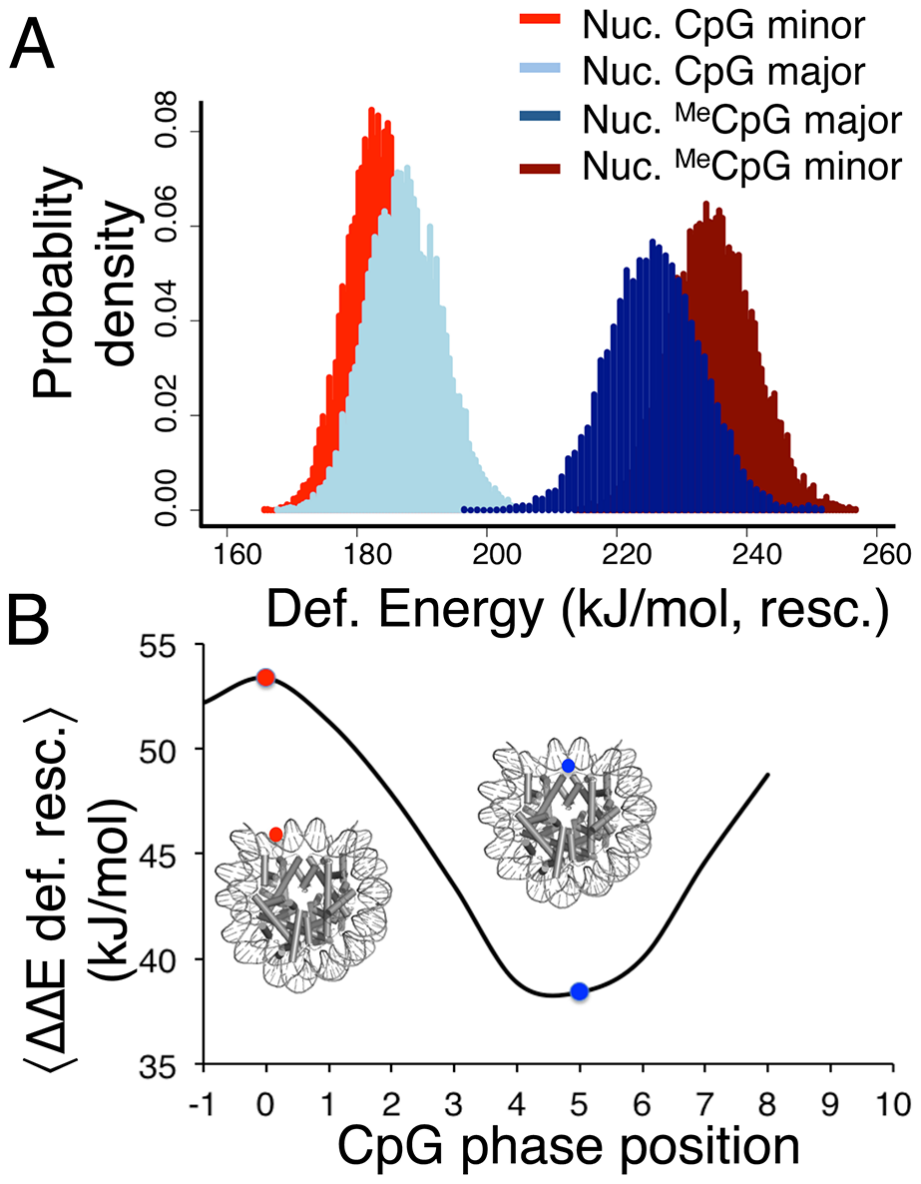
Methylated and non-methylated DNA elastic deformation energies. (A) Distribution of deformation energies for 147 bp-long random DNA sequences with CpG steps positioned every 10 base steps (one helical turn) in minor (red and dark red) and major (light and dark blue) grooves respectively. The energy values were rescaled by the slope of a best-fit straight line of figure 2, which is 0.23, to map the elastic deformation energies to ΔΔG. Methylation of the DNA causes a higher energetic cost for nucleosome formation, especially when the ^Me^CpG steps are positioned in the minor groove facing the histones (dark red). (B) Correlation between the additional energetic cost due to methylation (〈E_Meth_〉-〈E〉, kJ/mol) to form a nucleosome and the phasing of CpG steps respect to the histone (the zero is the reference phase position in which the CpG minor groove directly faces the histones). The cartoons illustrate two extreme positions of the methyl groups with respect to the histone core, which translate into a change in the rotational phase: the position of the methyl group, pointing to the solvent in 0 (red dot) and to the histones in 5 (blue dot).

Using the harmonic deformation method, we additionally predicted the change in stability induced by cytosine methylation for millions of different nucleosomal DNA sequences. Consistently with our calculations, we used two extreme scenarios to prepare our DNA sequences (see Fig. 3): i) all positions where the minor grooves contact the histone core are occupied by CpG steps, and ii) all positions where the major grooves contact the histone core are occupied by CpG steps. We then computed the elastic energy required to wrap the DNA around the histone proteins in un-methylated and methylated states, and, as expected, observed that methylation disfavors DNA wrapping (Figure 3A). We have rescaled the elastic energy differences with a factor of 0.23 to match the ΔΔG prediction in figure 2B. In agreement with the rest of our results, our analysis confirms that the effect of methylation is position-dependent. In fact, the overall difference between the two extreme methylation scenarios (all-in-minor vs all-in-major) is larger than 60 kJ/mol, the average difference being around 15 kJ/mol.

We have also computed the elastic energy differences for a million sequences with CpG/^Me^CpG steps positioned at all possible intermediate locations with respect to the position (figure 3B). The large differences between the extreme cases can induce rotations of DNA around the histone core, shifting its phase to allow the placement of the methylated CpG steps facing the histones through the major groove. It is illustrative to compare the magnitude of CpG methylation penalty with sequence dependent differences. Since there are roughly 1.5e88 possible 147 base pairs long sequence combinations (i.e., (4^n^+4^(n/2)^)/2, n = 147), it is unfeasible to calculate all the possible sequence effects. However, using our elastic model we can provide a range of values based on a reasonably large number of samples. If we consider all possible nucleosomal sequences in the yeast genome (around 12 Mbp), the energy difference between the best and the worst sequence that could form a nucleosome is 0.7 kj/mol per base (a minimum of 1 kJ/mol and maximum of around 1.7 kJ/mol per base, the first best and the last worst sequences are displayed in Table S3 in Text S1). We repeated the same calculation for one million random sequences and we obtained equivalent results. Placing one CpG step every helical turn gives an average energetic difference between minor groove and major groove methylation of 15 kJ/mol, which translates into ∼0.5 kJ/mol per methyl group, 2 kJ/mol per base for the largest effects. Considering that not all nucleosome base pair steps are likely to be CpG steps, we can conclude that the balance between the destabilization due to CpG methylation and sequence repositioning will depend on the sequence, and it appears that multiple minor groove methylations in a nucleosome are very likely to induce nucleosome repositioning.

**Figure 4:**
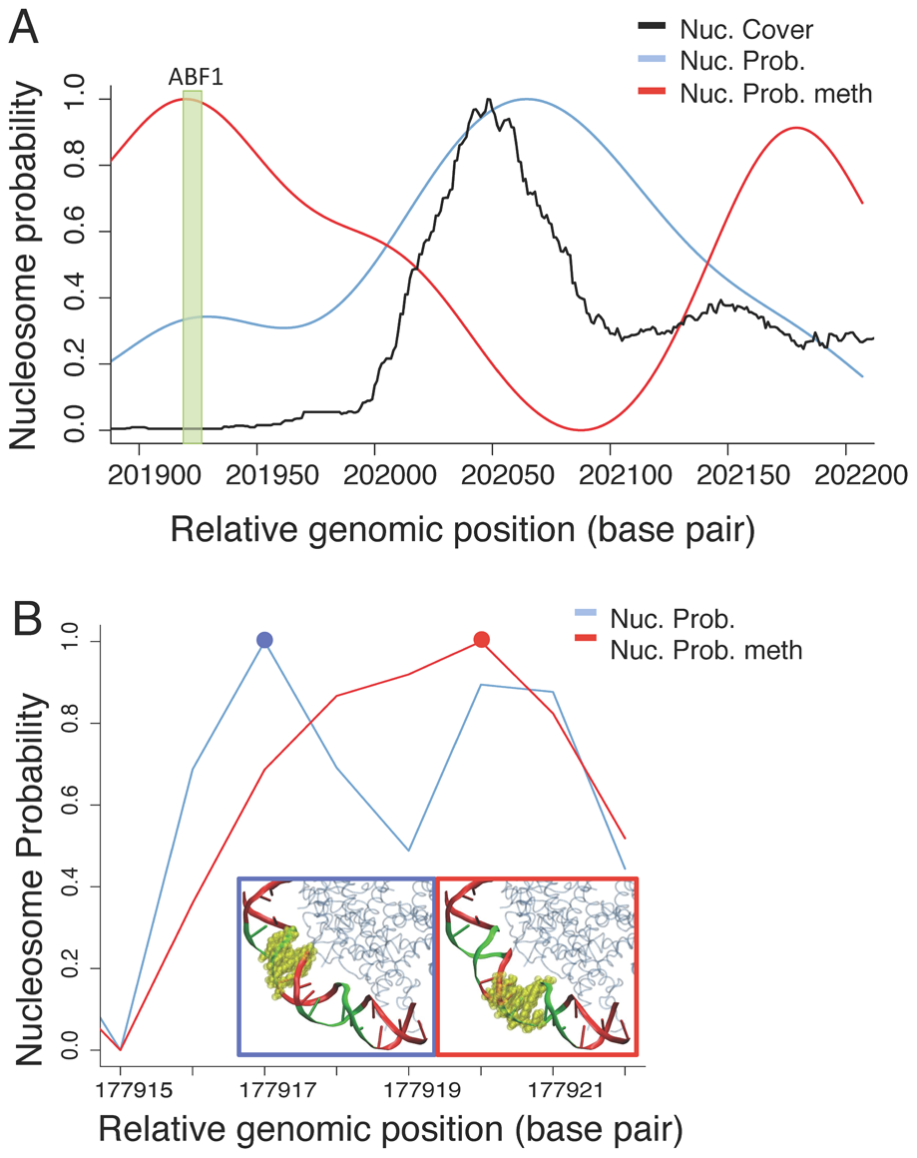
Examples of predicted impact of methylation on gene activity. (A) Predicted impact of methylation on the accessibility of transcription factors. The black line corresponds to the experimental population of nucleosomes (data from Deniz et al. [45]) in a portion of the yeast XV chromosome (close to the putative transcription start site of a gene, located at base pair 201879). The blue line represents the theoretical nucleosome probability, predicted from the elastic deformation energy for un-methylated DNA, and the red line shows the resulting nucleosome probability after CpG methylation. A vertical box highlights the binding position of the ABF1 transcription factor. (B) Example of the impact of methylation in nucleosome phasing. The blue line corresponds to the probability to wrap a nucleosome in a region of yeast chromosome VIII, where we have experimentally detected a stable nucleosome (Deniz et al. [45]) next to the PHD1 recognition box. The red line illustrates the nucleosome probability profile found when the sequence is methylated. We have depicted the associated change in translational positioning in the cartoons embedded in the figure: minor groove facing the histones in green, major groove facing the histones in red, and the PHD1 recognition box as yellow balls.

Changes in the phase of nucleosomal DNA could give rise to differences in gene activity, exemplified in figure 4 with two cases extracted from the yeast genome. We computed the relative probability to find a nucleosome centered in a given base pair using a Boltzmann-like probability distribution based on the differential elastic deformation energy. In the first example, figure 4A, both theory and experiment predict that the binding site of the transcription factor ABF1 (green box) is fully accessible. Upon CpG methylation, the predicted nucleosome probability curve changes (red line) and the histone core hides the ABF1 binding site. In figure 4B we show that methylation could induce a phase displacement that would change the accessibility of the recognition box of PHD1. Full details on these calculations can be found in the SI material. Both cases represented in these figures illustrate the impact of methylation in modulating binding of regulatory proteins to DNA by a simple chemical mechanism that affects nucleosome positioning.

In summary, the calculations reported here shed light on the physicochemical code behind epigenetic CpG methylation. State of the art calculations suggest that methylation disfavors nucleosome formation in a unique position-dependent manner, in agreement with recent experimental work [30], and that methylation induces changes in nucleosome positioning and phasing, resulting in a different pattern of well-positioned nucleosomes. This can change the accessibility of DNA to effector proteins and can affect then gene regulation. The present results also suggest a novel role for methylated DNA binding proteins: to keep the ^Me^C pointing towards the nucleosome exterior. Detachment of DNA binding proteins after methylation could lead to a spontaneous shift of the DNA's phase due to relaxation of the base steps towards more favorable positions. This relaxation modifies DNA accessibility and, accordingly, DNA read-out mechanisms. Overall our results support the existence of a basic physical code for the regulation of gene expression through chromatin organization. More complex mechanisms are probably built on top of it to define a fine control of the interplay between epigenetics, chromatin structure and gene regulation.

## Supporting Information

Text S1 **This file contains supporting methods; detailed description of supporting methods, algorithms and limitations.** Molecular dynamics simulations details, equilibration and mutation of the nucleosome models, mutations and thermodynamic integration method. Description of free energy calculation limitations, trajectory analysis, mesoscopic model of nucleosome deformation energy algorithm, mesoscopic model limitations rotational positioning and phase calculation. The supporting Tables S1, S2, S3 and the supporting Figures S1, S2, S3, S4, S5, S6, S7.
(DOCX)
