# Supplementary material for: Understanding the Connection between Epigenetic DNA Methylation and Nucleosome Positioning from Computer Simulations
Source: PLoS Comput Biol. 2013 Nov 21;9(11):e1003354. doi: 10.1371/journal.pcbi.1003354 (PMC3836855; doi:10.1371/journal.pcbi.1003354)
Supplement: Text S1 — This file contains supporting methods; detailed description of supporting methods, algorithms and limitations. Molecular dynamics simulations details, equilibration and mutation of the nucleosome models, mutations and thermodynamic integration method. Description of free energy calculation limitations, trajectory analysis, mesoscopic model of nucleosome deformation energy algorithm, mesoscopic model limitations rotational positioning and phase calculation. The supporting Tables S1, S2, S3 and the supporting Figures S1, S2, S3, S4, S5, S6, S7. (DOCX) [file pcbi.1003354.s001.docx]

**SUPPORTING INFORMATION**

**Understanding the connection between epigenetic DNA methylation and nucleosome positioning from computer simulations.**

Guillem Portella ^a,b‡^, Federica Battistini ^a,b‡^, Modesto Orozco ^a,b,c,*^.

a) Institute for Research in Biomedicine (IRB Barcelona), Barcelona, Spain

b) Joint IRB-BSC Program in Computational Biology, Barcelona, Spain

c) Departament de Bioquímica i Biologia Molecular, Universitat de Barcelona, Barcelona, Spain

* To whom correspondence should be addressed. Tel: +34934037156; Email: modesto.orozco@irbbarcelona.org

‡The authors wish it to be known that, in their opinion, the first two authors should be regarded as joint First Authors.

**Table of contents**

Supporting text: methods and references……………………………..… pg. 2-8

Supporting tables………………………………………………….. pg. 9-10

Supporting figures………………………………………………… pg. 11-18

**SUPPORTING METHODS**

**Supporting Text S1**

**Molecular dynamics simulations**

We performed molecular dynamics (MD) simulations and thermodynamic integration calculations for 21 different nucleosome sequences, 18 of them with single CpG steps and 4 cases with multiple CpG steps distributed along the sequence. A complete list of all simulations performed can be found listed in table 1. In the following sections we describe how we prepared, performed and analyzed these calculations.

**Equilibration and mutation of the nucleosome models**

We solvated the X-ray structure with PDB code 1KX5[1] (after removal of long histone tails protruding out from the core) in a truncated octahedron box containing 45000 water molecules, leaving 1.4 nm between the nucleosome atoms and the edges of the box. We added 14 manganese ions, 226 sodium ions and 34 chlorine ions to balance the nucleosome charge and give a realistic ionic atmosphere.

We generated the set of parameters describing the interactions for DNA and proteins based on the amber99SB[2]+parmBSC0[3] force fields, using the SPC/E[4] model to describe the water molecules. We took the parameters for sodium and chlorine ions from Smith *et al.*[5], and the manganese parameters from the Amber parameter database, and those parameters describing methylcytosine from Perez *et al*[6]. We minimized and then thermalized (T=300 K) the initial system during 10 ns. After that, we subjected the thermalized structure to 200 ns of MD simulation.

We used the last structure of the nucleosome from the 200 ns MD simulations as a template to introduce different number of CpG and methylated CpG steps in positions described in tables S1. After energy minimization and initial thermalization, we performed MD for 100 ns for the selected single mutations and 200 ns for the multiple mutations (see table 1 and the next section), gathering information concerning solvent interaction or solvent densities, energies of stacking and geometrical parameters.

We carried out all our MD simulations using the Gromacs-4.5 software [7], with periodic boundary conditions and the particle mesh Ewald [8] method for the long-range electrostatics, together with a cut-off of 1.0 nm for the short-range repulsive and attractive dispersion interactions, which were modeled via a Lennard-Jones potential. We used the Settle algorithm to constrain bond lengths and angles of water molecules, and P-Lincs[9] for all other bond lengths, allowing a time step of 2fs. We kept the temperature constant at 300 K by using the thermostat method of Bussi et al.[10] To control the pressure during the simulation we coupled the box to a pressure bath of 1 atm[11].

**Mutations and Thermodynamic Integration**

In table S1 we present a list of all mutations that were subject to thermodynamic integration calculations to establish the differential free energy of binding for methylated and un-methylated DNA. We introduced CpG in positions that face the protein core through the minor or, alternatively, the major grooves Such positions explore sites in the nucleosomal DNA of negative and positive opening of the base pairs along their long axis (the “roll” helical parameter, Suppl. Fig. S1A). The roll is a helical degree of freedom that is directly related to the overall bending of DNA [12]. Since CpG steps have a marked positive equilibrium roll value (+10 degrees) which increases upon methylation by +4 degrees [6], we expected the selected positions to be especially sensitive to cytosine methylation. In addition, we introduced several CpG steps to study multiple methylation effects on nucleosomal stability. For each mutation we initially performed 10 ns (20 ns for the multiple mutations) of MD simulations to accommodate the newly introduced CpG steps. For further analysis of the three distinct multiple mutations (minorGroove, majorGroove and Mixed1) we performed 200 ns of unrestricted MD for the methylated and un-methylated nucleosome containing systems, and 100 ns of MD for the systems containing single mutations (1 to 10) in the methylated and un-methylated state.

Since a direct calculation of the binding free energy is at the moment prohibitive, we instead use a thermodynamic cycle (Fig. 1B) and compute the reversible work associated to the alchemical transformation between two DNA sequences (in our case between methylated and un-methylated cytosines), both in the bound and in the unbound state. The details on how these mutations are carried out are outlined in the next paragraph. The calculations on the unbound reference state for the single mutations were performed on shorter DNA chains of 16 residues, using the nearest 3 neighbors of the CpG steps in the nucleosome sequence, and 4 bases to cap the duplex termini (5’-CGAT and TACG-3’). As the histone proteins are not affected by the cytosine methylation in the unbound state, they were not included in the calculations related to such state. For multiple mutations we divided the DNA in the unbound states in two relatively large fragments of different length (see table S2), each with 4 CpG steps, also flanked by 5’-CGAT and TACG-3’ termini. We performed 50 ns of MD for methylated and un-methylated duplexes used as reference state for the cases described in table 1. The reference systems were run in minimal salt conditions (to achieve charge neutrality) and we used the same force field and simulation conditions as for the nucleosomal systems.

To achieve the aforementioned alchemical transformation between methylated and un-methylated cytosines, our molecular dynamics simulations convert the methyl group in position 5 of the cytosine ring into a hydrogen atom (or *vice versa*). This transformation is achieved by parameterizing the Hamiltonian $\mathcal{H}$ that describes the systems as function of a coupling parameter. The coupling parameter defines whether we are in the methylated, un-methylated or mixed (non-physical) states. Since the free energy is a state function, the value of the free energy difference is independent on the chosen path. We used the thermodynamic integration method in its discrete formalism (DTI), where the free energy between two states is λ=0 and λ=1 is computed by

$${\Delta G}_{0\to1}=\int_{0}^{1} \left\langle\frac{\partial\mathcal{H}}{\partial\lambda} \right\rangle_{\lambda}d\lambda$$

, i.e. by integration of the derivative of the energy of the system as function of the state parameter λ, known as coupling parameter [13].

For each mutation, the variation of λ is discretized in 21 windows, i.e. dλ=0.05, and the final ΔG is computed via numerical integration. We used soft-core potentials implemented in Gromacs to avoid singularities in the Lennard-Jones and Coulomb potentials, with α=0.3, σ=0.25 and a soft-core power of 1. The initial structures for each window were obtained from a 10 ns simulation in which λ was continuously varied from 0 to 1. From this simulation we extracted frames corresponding to a given λ value, and we relaxed the structures by minimizing the energy of the system in those configurations. We simulated each window at fixed λ value for 1 ns, and we discarded the first 100 ps of simulation. For each window we collected 9 estimates for $\left\langle{\delta\mathcal{H}}/{\delta\lambda} \right\rangle_{\lambda}$ by using 9 blocks of 100 ps, which were then integrated through the entire mutation pathway to obtain mutation free energies (with associated statistical errors).

**Free energy calculation limitations**

MD/DTI is a rigorous theoretical approach that can be used to determine the changes in binding free energy induced by alterations in the chemical structure of a system. Its accuracy is limited by that of the force field (taken for granted) and by the length of the trajectory, that should be large enough to capture any possible conformational transition related to the mutation. Since in this study these conformational changes are small, we can assume MD/DTI estimates should be accurate enough to extract meaningful conclusions (see also the statistical error in table S1).

**Trajectory analysis**

From our MD simulations we extracted water densities around methylated and un-methylated cytosines using a cubic grid of size of 0.05 nm, both for the duplex reference state and the nucleosome particle. We used the *g_rdf* tool provided in the Gromacs suite of programs to compute the radial distribution function of solute molecules around the nucleic acids and quantify the number of water molecules within 0.6 nm of the cytosine ring center. We computed also the time averaged stacking and base pairing interaction energies between the bases in the CpG steps and their first nearest neighbors, methylated and un-methylated. As staking interactions we included the short-range Coulomb and Lennard-Jones interaction energies between nucleotides of consecutive steps, whereas the base pairing interactions considered the nucleotides forming Watson-Crick base pairs. In Figure S4 we displayed the computed differences between these interactions in presence and absence of a methylated cytosine, both in the nucleosomes and in the duplex reference state. Time averaged helical parameters were extracted from the molecular dynamics trajectories with a frequency of 100 ps using the program Curves+ [14].

**Mesoscopic model of nucleosome deformation energy**

Physical and geometrical descriptors derived from molecular dynamics (MD) simulations were used to study DNA deformability at the base-pair-step level, and to evaluate nucleosome energy formation. Instead of using six Cartesian coordinates, the geometry of two consecutive base pairs, a DNA base-pair-step, can be described with a set of six helical movement parameters (so-called *helical parameters*): three translations (rise (s), slide (l) and shift (f)) and three rotations (twist (w), roll (r), tilt (t)). The deformability along those movements can be described by the stiffness constants (*k_i_*) associated with the displacements with respect to the equilibrium values of the helical parameters [15,16]. The values for the parameters describing the equilibrium geometry and stiffness constants of naked DNA were derived from long atomistic MD simulations (>200ns, as found in the ABC consortium database[17]) of a reduced number of short DNA duplexes in water. The parameters for methylated cytosine were extracted from Perez *et al.*[6]. To obtain the equilibrium values and associated force constants of each helical parameter for each different base pair step, we projected the DNA geometries extracted from the MD simulations into a helical reference system. By collecting the time-averaged values of these helical parameters we built a covariance matrix for each unique base pair step. The inversion of this covariance matrix allows the determination of elastic force constants. The energy associated to a given base pair deformation was computed using a harmonic approximation, given by


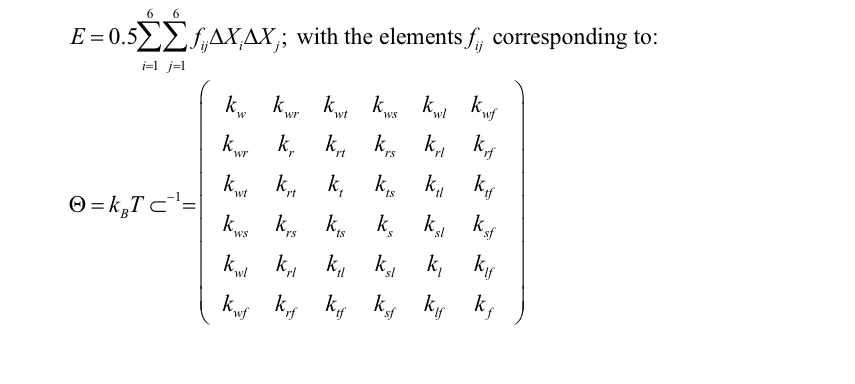


where *k_b_* is the Boltzmann constant, *T* is the absolute temperature, *E* is the energy associated to the given deformation*,* and *k* stands for the different stiffness constants defining by the 36 elements of the stiffness matrix (**Θ**) at the dinucleotide level (in different tetramer environments) [15,16].

We measured the impact of the CpG methylation on the ability of DNA to wrap around a histone octamer via deformation energy for the methylated and un-methylated sequences, using the mesoscopic energy model just described. For each sequence, the difference in elastic energy of the DNA in the nucleosome with respect to the unbound state was computed by considering the helical parameters that each base pair adopts in the MD-averaged conformation (described in the Equilibration section of this SI material). Since we know the equilibrium values and the associated force constants for each base pair step in free solution, we can calculate the energy required to wrap a 147 base pair long DNA sequence into a nucleosome conformation using our harmonic approximation. By performing this operation for the methylated and un-methylated sequence, we can calculate the differences in elastic deformation energy due to CpG methylation,

ΔΔE def. = ΔE def. methylated – ΔE def. un-methylated

We have also used the elastic deformation energy as descriptor to analyze the effect of methylation on randomly generated sequences. To that end, we generated one million of random sequences 147 base pairs long with CpG steps placed every 10 bases. We initially considered two extreme cases in which all the CpG steps are place at positions where the minor groove or the major groove faces the histones. The selectivity of groove placement was possible based on the MD-averaged nucleosome structure. We calculated the deformation energy to wrap every sequence around the histones, with and without CpG methylation. For each of the four cases analyzed (CpGs/^Me^CpGs with minor or major groove facing the histones), we represented the probability density of their deformation energies as a histogram in Figure 3A. We have repeated these calculations for CpG steps located at intermediate positions of their grooves with respect to the histones. This was achieved by incrementally shifting all the position of these CpG steps by one base along the sequences until we completed a helical turn, retaining a 10 base pair separation between them. This shift in the CpG positions along the sequence effectively explores the phase changes of groove positioning with respect to the histones. We averaged the differential deformation energy due to CpG methylation over all the sequences for each CpG phase position. The averaged differential deformation energy as function of the CpG phase position is represented in figure 3B.

We also characterize the effect of CpG methylation on the nucleosome occupancy in two regions of the yeast genome using our elastic energy model. From *in vitro* MNase digestion experiments (data from Deniz *et al.* [18]) we obtained the nucleosome population distribution surrounding the TSS of chromosome XV (location at base pair 201879) in yeast genes. The binding site of the transcription factor ABF1 is situated in this chromosome location, in a nucleosome-free region upstream from a well-positioned nucleosome. Using the difference in elastic deformation energy for nucleosome formation, we used the Boltzmann-like probability distribution, exp(-ΔE_def_/*k_b_*T), to determine the relative probability to position a nucleosome on a given base pair. We repeated the same probability calculations for the same sequences but now with methylated CpG steps. In order to compare the theoretically determined nucleosome localization probabilities with the experimentally determined nucleosome coverage (also a measure of nucleosome localization probability) we have re-scaled all probability values from 0 to 1. The results of this comparison can be found in figure 4A.

Finally, we studied the impact of methylation on the location of the transcription factor binding-box of PHD1. Using the approach presented in the previous paragraph, we calculated the nucleosome localization probability for the genome segment of the yeast chromosome VIII (location 177908-178032), with and without CpG methylation (figure 4B). We calculated the distance between the binding box of the transcription factor PHD1 and the dyad of the nearest most probable nucleosome according to our calculations. We then compared the value of this distance for the un-methylated and methylated nucleosomes (blue and red dots on the probability profiles respectively). Using these distances, we could determine that the predicted translational repositioning of nucleosome profiles by 3 base pairs upon CpG methylation implies a change in accessibility of the recognition site of PHD1, moving the minor groove away from the histones towards the solvent (this repositioning is represented in the embedded nucleosome cartoons in Figure 4B).

**Mesoscopic model limitations**

Elastic calculations assume linearity between the force and the displacement for each deformation and, accordingly, are valid just for small to moderate deformations (like those found in nucleosomes). Here we also assume that the magnitude of stabilizing histone-DNA interactions is sequence independent and that the geometry of DNA around the histone core is that found in the MD-averaged structures (this is not necessarily the case for the calculations regarding the random sequences and that were not simulated by MD). All these are severe assumptions, which means that elastic estimates need to be validated against experimental data or high-level MD/DTI results, as done here.

**Rotational Positioning and Phase Calculation**

We calculated the contribution of an individual base step to the global curvature in the nucleosomal DNA before and after methylation. The curvature was determined from the rotational local base step parameters roll (r) and tilt (t), and their contributions to the global curvature were modulated by the sine and the cosine of the cumulative helical twist relative to the dyad, Φ*n*. For a complete derivation of this geometrical descriptor we refer to Battistini *et al* [19], where it was shown that the curvature given by the roll is the major component for the bending of the DNA around the histones, a result that was validated here. Thus, in this work we used Φ*n* to describe the curvature of the DNA around the histone core. In order to examine DNA curvature in detail, before and after methylation, we compare the two rotational settings (Suppl. Figure S7A). First of all, we calculated the curvature over the whole sequences, given by roll as a cosine function of the helical phase, and a possible phase offset between the two cases. The optimal helical phase is the one that maximizes the roll curvature contribution respect to the others. This optimization can be applied because the bending is almost entirely on one plane. When DNA is un-methylated the optimal helical phase is given by the cumulative helical twist along the sequence. Interestingly, when all the methylations were located at positions where the minor groove faces the histone core we detected that we would have to modify the helical phase adding a phase offset of 20-30 degrees to match the roll component of the bending for the un-methylated DNA. This is, to match the un-methylated local bending profile after methylation in the minor groove positions we have to rotate the DNA. This rotation implies displacing the rotational setting by shifting the dyad position of almost one step. The rotational re-positioning is a small movement (as seen in Suppl. Figure S7B) and can be noticed in particular in the CpG step close by the dyad.

**References for supplementary methods**

1. Davey CA, Sargent DF, Luger K, Maeder AW, Richmond TJ (2002) Solvent mediated interactions in the structure of the nucleosome core particle at 1.9 a resolution. Journal of molecular biology 319: 1097–1113. Available: http://dx.doi.org/10.1016/S0022-2836(02)00386-8. Accessed 14 November 2012.

2. Hornak V, Abel R, Okur A, Strockbine B, Roitberg A, et al. (2006) Comparison of multiple Amber force fields and development of improved protein backbone parameters. Proteins 65: 712–725. Available: http://www.ncbi.nlm.nih.gov/entrez/query.fcgi?cmd=Retrieve&db=PubMed&dopt=Citation&list_uids=16981200.

3. Pérez A, Marchán I, Svozil D, Sponer J, Cheatham TE, et al. (2007) Refinement of the AMBER force field for nucleic acids: improving the description of $\alpha$/$\gamma$ conformers. Biophysical journal 92: 3817–3829. Available: http://linkinghub.elsevier.com/retrieve/pii/S0006349507711827.

4. Berendsen HJC, Grigera JR, Straatsma TP (1987) The missing term in effective pair potentials. The Journal of Physical Chemistry 91: 6269–6271. Available: http://pubs.acs.org/doi/abs/10.1021/j100308a038. Accessed 20 May 2011.

5. Smith DE, Dang LX (1994) Computer simulations of NaCI association in polarizable water. 99352: 3757–3766.

6. Pérez A, Castellazzi CL, Battistini F, Collinet K, Flores O, et al. (2012) Impact of Methylation on the Physical Properties of DNA. Biophysical Journal 102: 2140–2148. Available: http://dx.doi.org/10.1016/j.bpj.2012.03.056. Accessed 14 July 2012.

7. Hess B, Kutzner C, van der Spoel D, Lindahl E (2008) GROMACS 4:&nbsp; Algorithms for Highly Efficient, Load-Balanced, and Scalable Molecular Simulation. Journal of Chemical Theory and Computation 4: 435–447. Available: http://pubs.acs.org/doi/abs/10.1021/ct700301q.

8. Darden T, York D, Pedersen L (1993) Particle mesh {E}wald: an {N}$\\cdot$log({N}) method for {E}wald sums in large systems. J Chem Phys 98: 10089–10092.

9. Hess B (2008) P-LINCS: A Parallel Linear Constraint Solver for Molecular Simulation. J Chem Theory Comput 4: 116–122.

10. Bussi G, Donadio D, Parrinello M (2007) Canonical sampling through velocity rescaling. The Journal of chemical physics 126: 014101+.

11. Berendsen HJC, Postma JPM, DiNola A, Haak JR (1984) Molecular dynamics with coupling to an external bath. J Chem Phys 81: 3684–3690.

12. Calladine CR, Drew HR, Luisi BF, Travers AA (2004) Undertanding DNA. The Molecule and how it works. Third. San Diego, California: Elsevier Academic Press.

13. Kirkwood JG (1935) Statistical mechanics of fluid mixtures. J Chem Phys Phys 3: 300–313.

14. Lavery R, Moakher M, Maddocks JH, Petkeviciute D, Zakrzewska K (2009) Conformational analysis of nucleic acids revisited: Curves+. Nucleic Acids Research 37: 5917–5929.

15. Lankas F, Sponer J, Langowski J, Cheatham  3rd TE (2003) DNA basepair step deformability inferred from molecular dynamics simulations. Biophysical Journal 85: 2872–2883.

16. Olson WK, Gorin AA, Lu XJ, Hock LM, Zhurkin VB (1998) DNA sequence-dependent deformability deduced from protein-DNA crystal complexes. Proceedings of the National Academy of Sciences of the United States of America 95: 11163–11168.

17. Lavery R, Zakrzewska K, Beveridge D, Bishop TC, Case DA, et al. (2010) A systematic molecular dynamics study of nearest-neighbor effects on base pair and base pair step conformations and fluctuations in B-DNA. Nucleic acids research 38: 299–313. Available: http://nar.oxfordjournals.org/content/38/1/299. Accessed 15 November 2012.

18. Deniz O, Flores O, Battistini F, Pérez A, Soler-López M, et al. (2011) Physical properties of naked DNA influence nucleosome positioning and correlate with transcription start and termination sites in yeast. BMC genomics 12: 489. Available: http://www.pubmedcentral.nih.gov/articlerender.fcgi?artid=3224377&tool=pmcentrez&rendertype=abstract. Accessed 18 March 2013.

19. Battistini F, Hunter CA, Gardiner EJ, Packer MJ (2010) Structural mechanics of DNA wrapping in the nucleosome. Journal of molecular biology 396: 264–279. Available: http://dx.doi.org/10.1016/j.jmb.2009.11.040. Accessed 22 November 2012.

| Mutation Index  or  mutation type | Position of the CpG steps with respect to the nucleosome dyad | | Position of the first base pair along the nucleosomal sequence | Groove position  facing the histones | Differential binding free energy (kJ/mol) |
| --- | --- | --- | --- | --- | --- |
| 1 | 0.5 | | 74 | Major | 4.9 +/- 0.6 |
| 2 | -35.5 | | 38 | Minor | 0.7 +/- 0.6 |
| 3 | -29.5 | | 44 | Major | 1.6 +/- 0.8 |
| 4 | -19.5 | | 54 | Major | 4.3 +/- 0.9 |
| 5 | -16.5 | | 57 | Minor | 9.6 +/- 0.6 |
| 6 | -10.5 | | 63 | Major | -0.1 +/- 0.9 |
| 7 | -0.5 | | 73 | Major | 0.0 +/- 0.8 |
| 8 | 4.5 | | 78 | Minor | 4.5 +/- 0.6 |
| 9 | 20.5 | | 94 | Major | 0.7 +/- 0.9 |
| 10 | 26.5 | | 100 | Minor | 11.1 +/- 1.0 |
| 11 | 35.5 | | 109 | Minor | 4.9 +/- 1.8 |
| 12 | -11.5 | | 62 | Major | -0.8 +/- 0.9 |
| 13 | -6.5 | | 67 | Minor | 5.1 +/- 0.9 |
| 14 | 8.5 | | 82 | Major | 5.4 +/- 1.4 |
| 15 | 16.5 | | 90 | Minor | -1.3 +/- 1.0 |
| 16 | -26.5 | | 47 | Minor | 11.1 +/- 0.8 |
| 17 | -41.5 | | 32 | Major | -0.3 +/- 0.8 |
| 18 | 29.5 | | 103 | Major | 3.3 +/- 0.7 |
|  |  | |  |  |  |
| MinGroove | | -35.5 | 38 | Minor | 35.0 +/- 2.2 |
|  | | -26.5 | 47 | Minor |  |
|  | | -16.5 | 57 | Minor |  |
|  | | -6.5 | 67 | Minor |  |
|  | | 4.5 | 78 | Minor |  |
|  | | 16.5 | 90 | Minor |  |
|  | | 26.5 | 100 | Minor |  |
|  | | 35.5 | 109 | Minor |  |
| MajGroove | | -41.5 | 32 | Major | 7.8 +/- 3.6 |
|  | | -29.5 | 44 | Major |  |
|  | | -19.5 | 54 | Major |  |
|  | | -11.5 | 62 | Major |  |
|  | | 8.5 | 82 | Major |  |
|  | | 20.5 | 94 | Major |  |
|  | | 29.5 | 103 | Major |  |
|  | | 36.5 | 114 | Major |  |
| Mixed1 | | -19.5 | 54 | Major | 15.6 +/- 2.1 |
|  | | -16.5 | 57 | Minor |  |
|  | | -11.5 | 62 | Major |  |
|  | | -6.5 | 67 | Minor |  |
|  | | 4.5 | 78 | Minor |  |
|  | | 8.5 | 82 | Major |  |
|  | | 16.5 | 90 | Minor |  |
|  | | 20.5 | 94 | Major |  |
| Mixed2 | | -33.5 | 40 | ~Minor | 25.4 +/- 1.4 |
|  | | -25.5 | 48 | ~Minor |  |
|  | | -13.5 | 60 | ~Major |  |
|  | | -4.5 | 69 | ~Minor |  |
|  | | 2.5 | 76 | ~Major |  |
|  | | 17.5 | 91 | ~Minor |  |
|  | | 28.5 | 102 | ~Major |  |
|  | | 43.5 | 117 | ~Major |  |

**Supplementary Table S1.** **List of the single and multiple mutations.** Mutations introduced exchanging the original base pair steps for CpG base pair steps) of the DNA sequence 1KX5 for every case studied, indicating the position of the mutation relative to the nucleosome dyad, the corresponding position of the first base pair along the nucleosome sequence, and the orientation of the grooves with respect to the histone core. In the last column we list the corresponding differential binding free energy with respect to the un-methylated sequence obtained in this work by means of DTI. In Mixed2, the CpG step grooves are not perfectly co-planar with respect to the histone core; the groove side that is closer to the surface of the histone core has been specified with the symbol ~.

| Fragment | Sequence (5’ to 3’) |
| --- | --- |
| Mut-1 | CGATGGA**CG**CCATACG |
| Mut-2 | CGATATT**CG**GAATACG |
| Mut-3 | CGATAAA**CG**GCTTACG |
| Mut-4 | CGATATC**CG**AAGTACG |
| Mut-5 | CGATAAA**CG**GCATACG |
| Mut-6 | CGATCAT**CG**TCATACG |
| Mut-7 | CGATTGG**CG**TCCTACG |
| Mut-8 | CGATTCC**CG**CTGTACG |
| Mut-9 | CGATTTT**CG**ATGTACG |
| Mut-10 | CGATTTT**CG**ATGTACG |
| Mut-11 | CGATTTC**CG**AATTACG |
| Mut-12 | CGATGCA**CG**TTCTACG |
| Mut-13 | CGATTTC**CG**CTGTACG |
| Mut-14 | CGATGCT**CG**ACATACG |
| Mut-15 | CGATTGC**CG**TTTTACG |
| Mut-16 | CGATCTG**CG**CCATACG |
| Mut-17 | CGATAAG**CG**TATTACG |
| Mut-18 | CGATAGC**CG**TTTTACG |
| Minor part 1 | CGATATT**CG**GAAACTG**CG**CCATCAAA**CG**GCATGTTC**CG**CTGTACG |
| Minor part 2 | CGATTCC**CG**CTGAACATGC**CG**TTTGATGG**CG**CAGTTTC**CG**AATTACG |
| Major part 1 | CGATAAG**CG**TATTTGGAAA**CG**GCTCCATC**CG**AAGGCA**CG**TTCTACG |
| Major part 2 | CGATGCT**CG**ACATGCCTTT**CG**ATGGAGC**CG**TTTCCAAAT**CG**ACTTACG |
| Mixed1 part 1 | CGATATC**CG**A**CG**GCA**CG**TTC**CG**CTGTACG |
| Mixed1 part 2 | CGATTCC**CG**CT**CG**ACATGC**CG**TT**CG**ATGTACG |

**Supplementary Table S2.** **List of all sequences used as reference for the free energy calculations.** The positions of the CpG steps are highlighted in bold letters.

| Fragment | Sequence (5’ to 3’) |
| --- | --- |

| Sequence | ΔE elastic def. (kJ/mol) |
| --- | --- |
|  | |
| CACTAAATCGTCGAAGAGCTTCTTGACCGTTTTTATCCGAAGTGTTTTTCCTCTTTCCCCAAGTTTTCCTGCAGGCGGGGGAATATGGGGCCCCATGGAAAAAAAACCGGGGGGGGTGGGAAAAAAAAAGGGGTCAGAAAAAAAAAC | 153 |
| TCGAAGAGCTTCTTGACCGTTTTTATCCGAAGTGTTTTTCCTCTTTCCCCAAGTTTTCCTGCAGGCGGGGGAATATGGGGCCCCATGGAAAAAAAACCGGGGGGGGTGGGAAAAAAAAAGGGGTCAGAAAAAAAAACTGCTCAGGGA | 154 |
| GCACTAAATCGTCGAAGAGCTTCTTGACCGTTTTTATCCGAAGTGTTTTTCCTCTTTCCCCAAGTTTTCCTGCAGGCGGGGGAATATGGGGCCCCATGGAAAAAAAACCGGGGGGGGTGGGAAAAAAAAAGGGGTCAGAAAAAAAAA | 155 |
| GATGGCGAAAACTTTGGCACAAGGAAGGAAACCTGGAAGCGGCAGAAAGCCCGGAAAAGGGAAGACGTTGAGAGAGGGAAGAAAGCCTGGCAGTGGTAGGAGGAGGAGGCAAGATACTGGGGGTAAAGAGACCGACGGGTCTCAGCA | 155 |
| … | |
| AATTATATAAAAATTATTAAATAAATATATAATATATTATATATAATTTATAATATATATATTATAAATATTATTATATATAAAATATAATATACTACTTATAAAAATATATATATATATATAAATATATATATAAATAAATATTTT | 254 |
| ATTAATTAATTAATAATATAAAAATATATATTATATATTATGTTTTATTTATATATATATATATATTATGTATTATTATATAAATATATATATATATTATATTATAAGTAATAATAAGTATTATATTATATATAGCTTTTATAGCTT | 254 |
| TAATTATATAAAAATTATTAAATAAATATATAATATATTATATATAATTTATAATATATATATTATAAATATTATTATATATAAAATATAATATACTACTTATAAAAATATATATATATATATAAATATATATATAAATAAATATTT | 254 |
| AAAATTATTAAATAAATATATAATATATTATATATAATTTATAATATATATATTATAAATATTATTATATATAAAATATAATATACTACTTATAAAAATATATATATATATATAAATATATATATAAATAAATATTTTATATATTAA | 255 |

**Supplementary Table S3.** **List of the first best and the last worst nucleosome forming sequences found in the yeast genome according to our elastic energy model.** These energy values are computed using as references the equilibrium values for the helical degrees of freedom of naked DNA, and only the relative differences between the reported numbers have physical meaning.


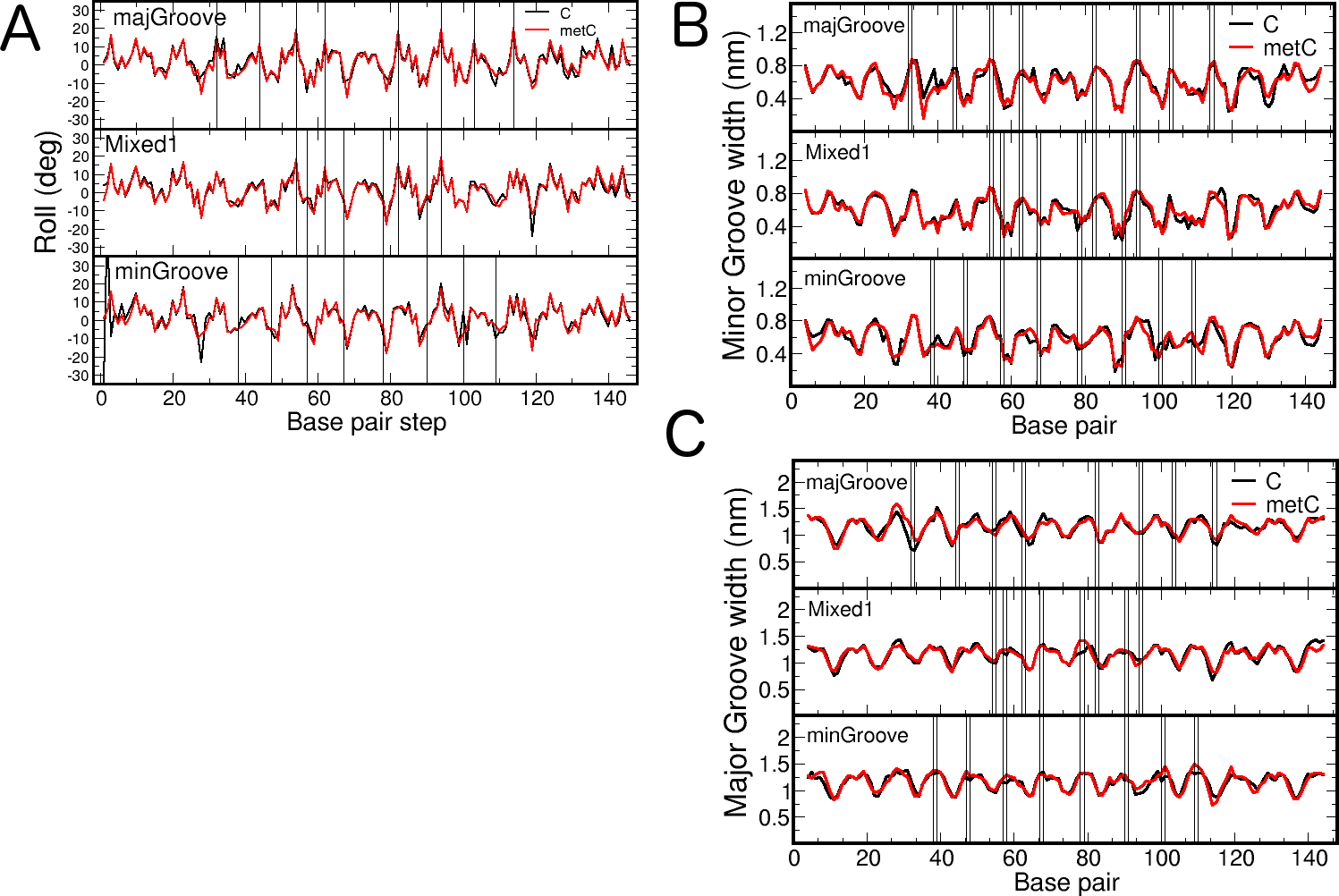


**Supplementary Figure S1.** **Base pair roll angle and groove width for three selected cases of methylated and un-methylated nucleosomal DNA.** (A) Roll angle of base pair steps in nucleosomal DNA for three extreme cases of multiple mutations, averaged over a MD trajectory of 100 ns. The curves in black correspond to systems with un-methylated CpG steps, whereas the red curves describe the helical parameter for methylated CpG steps. Vertical lines indicate the position of the CpG steps along the sequence. Notice that majGroove positions correspond to regions of high positive roll, whereas minGroove positions correspond to regions of negative roll. (B and C) Minor and major groove width of the nucleosomal DNA, averaged over 100ns MD simulation, for three different cases of multiple methylations. The curves in black show the values corresponding to methylated DNA; the red curves display the results for un-methylated DNA. The vertical lines indicate the position of the methylated CpG steps. The statistical error associated to the mean is around 0.3 nm, actually larger than the small variations observed between the methylated and un-methylated groove profiles.


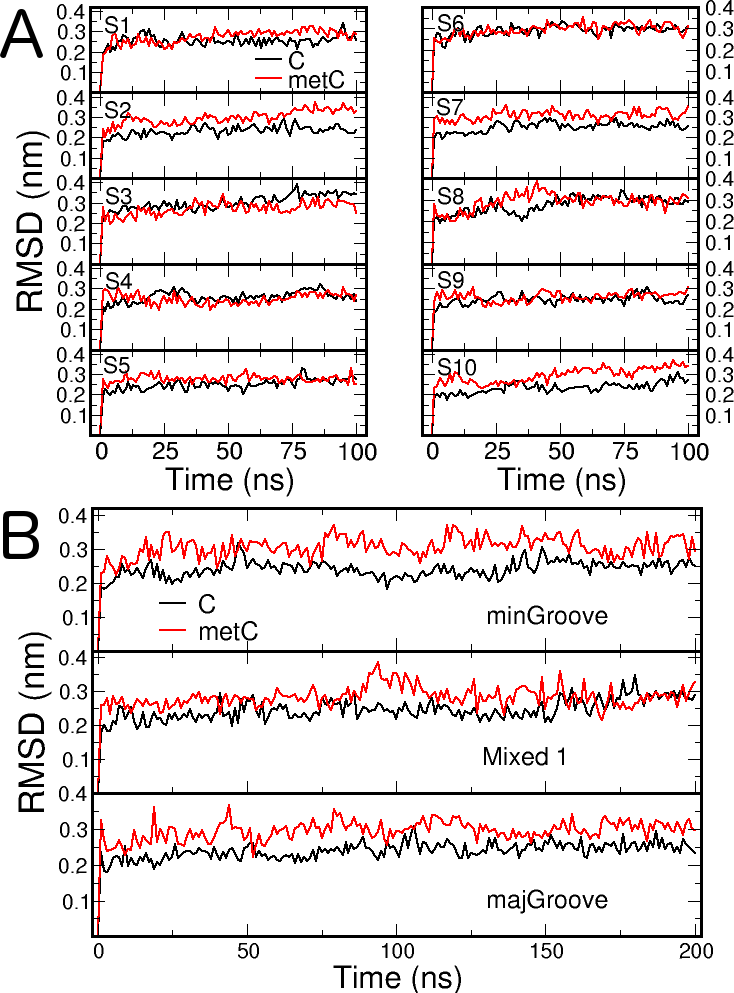


**Supplementary Figure S2**. **Time dependent root mean square deviation (RMSD) of all DNA atoms in selected nucleosome mutations.** (A) RMSD for the first 10 single nucleosome mutations presented in this work and (B) for three extreme cases of multiple nucleosome methylations. The RMSD were computed taking as reference the initial structure after short MD simulation to allow for relaxation of the modified structure. Black curves indicate the RMSD values corresponding to un-methylated derivatives, and the red curves show the results for the CpG-methylated nucleosomes.

**
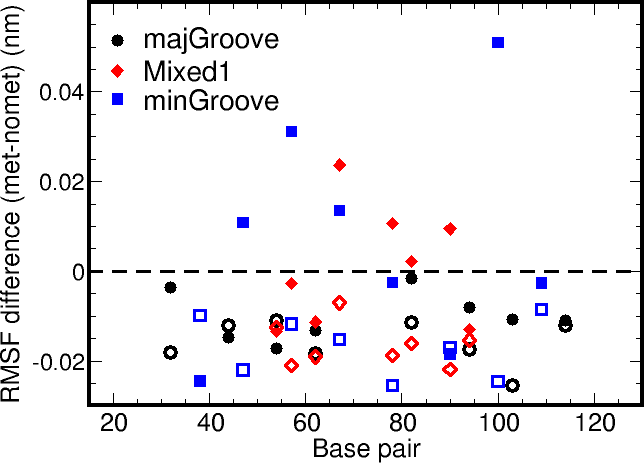
**

**Supplementary Figure S3.** **Averaged differences in methylated and un-methylated root mean square fluctuation (RMSF).** RMSF for the tetramer containing the CpG steps in the nucleosome (filled symbols) and in the reference naked DNA (empty symbols), obtained from our MD simulations. The average is performed over the tetramers containing the CpG steps in the central part, and the RMSF value is associated to the first C·G base pair along the nucleosome. The base pair number in the reference naked DNA was mapped to match the numeration of the nucleosome base pairs. Note that the fluctuations of the CpG steps in the naked reference state (empty symbols) are smaller for the methylated base pairs, indicating greater stiffness of these base pairs. Upon nucleosome formation the fluctuations around their mean position tends to increase for the methylated base pairs, especially for the most unfavorable positions (Mixed1 and minGroove, filled symbols).


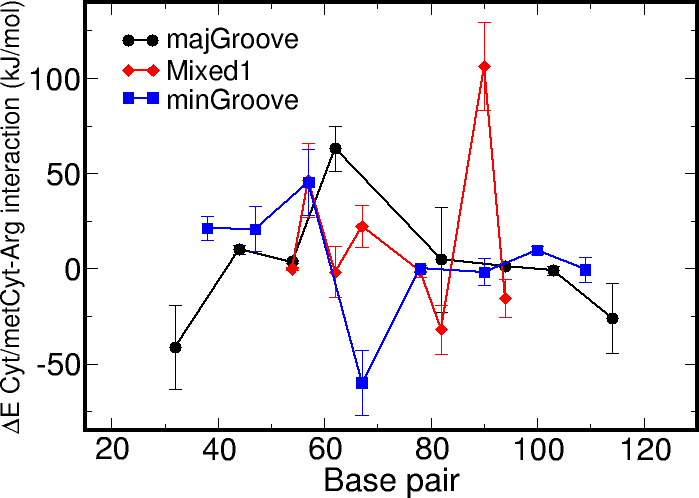


**Supplementary Figure S4.** **Time averaged difference in interaction energies between CpG tetramers and arginines.** Interaction energies calculated between the tetramers containing the methylated CpG step and the arginines residues from the histone core with respect to the situations where these CpG steps were un-methylated. The interaction energies shown are the time-averaged potential energy between the tetramers and the arginine residues, which can be understood as an enthalpic contribution. In the force-field approximation used in this work these potential energies are accounted for by a short-range Coulomb (electrostatic) term and a Lennard-Jones term (the later avoids atomic overlap and represents dispersion interactions). The differences in interaction energies (y-axis) are associated to the first C·G base pair along the nucleosome (x-axis). The pattern of differential interaction energies does not correlate with the changes in nucleosome stability predicted by means of free energy differences, e.g. the interaction energies are not consistently less favourable for the minGroove methylations than for the majGroove methylations, contrary to what has been observed for the overall binding free energy.

**
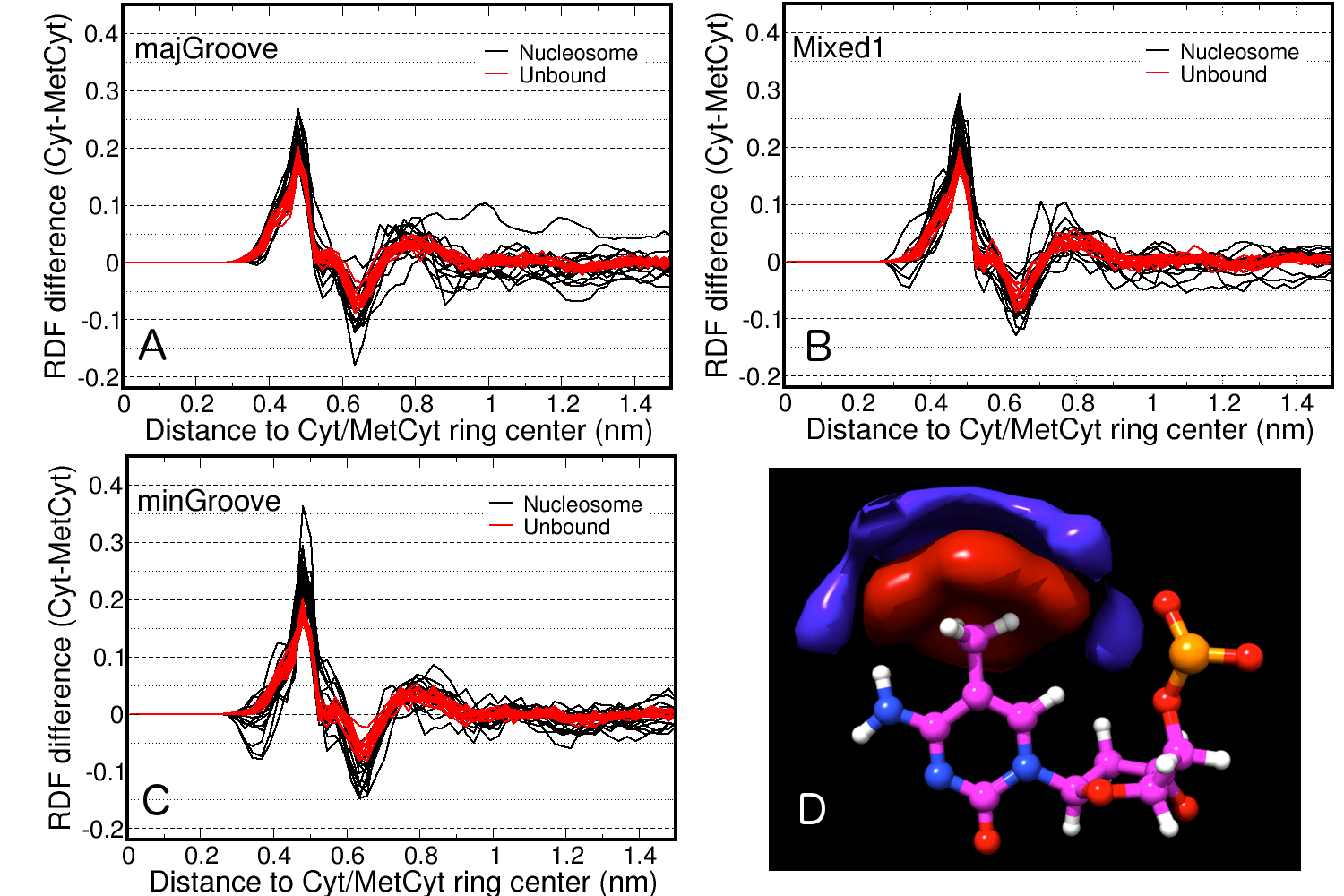
**

**Supplementary Figure S5.** **Differences in water radial distribution function and density.** (A to C) Differences in water radial distribution function between un-methylated and methylated cytosines in all the CpG steps for the multiple mutation cases. The center of the cytosine ring is taken as a reference and each individual curve corresponds to one methylated/un-methylated cytosine in a CpG step. The black curves correspond to histone-bound DNA; the red curves show the solvation differences in the case of naked DNA. Notice that in both extreme cases (A and C), where all mutations happen either where the major groove or the minor groove faces the histone core, the differences in water solvation are fairly similar. The differences in water solvation up to a cut-off length of 0.6 are only of a fraction of a water molecule. Furthermore, this value does not strongly depend on the location of the CpG steps, since even in the case where the methyl group points towards the histone core (majGroove) the presence of water pockets between histones and DNA ensures good short-range solvation of the methyl groups, similar to the minGroove placement. In panel C there is a slight increase of water solvation for methylated cytosines at around 0.35 nm due a slight rearrangement of the phosphate group, but this does not translate into a significant solvation difference up to a cut-off of 0.6 nm. (D) Difference in water density around a methylated/un-methylated cytosine in unbound DNA, in analogy with curves A to C. The red volume shows a region of high-water density in the un-methylated cytosine, the blue volume shows where the density of water is higher for the methylated cytosine. As a consequence of the excluded volume exerted by the methyl group, the water solvation is pushed away form the cytosine ring upon methylation.

**
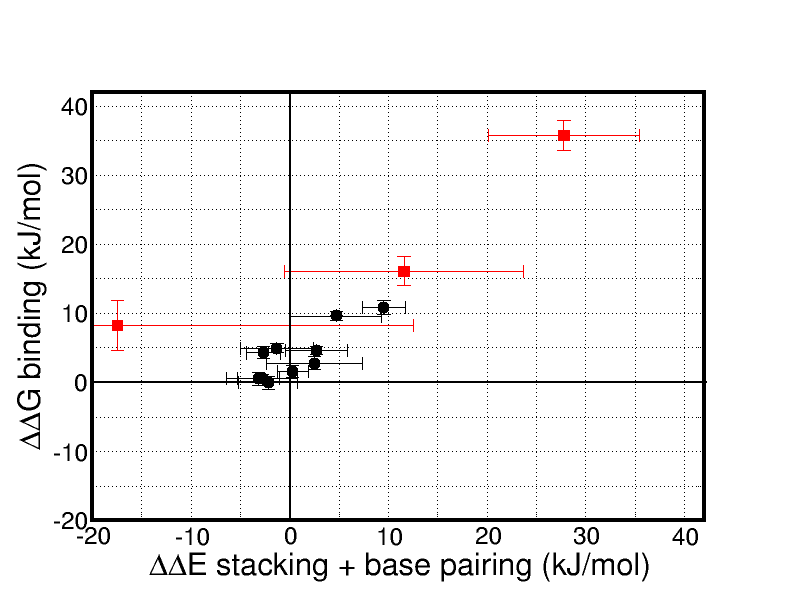
**

**Supplementary Figure S6**. **Correlation between nucleosome (de)-stabilization upon methylation (differential binding free energy, ΔΔG, y-axis) and the associated energy penalty for the modified base pairs (ΔΔE stacking + base pairing, x-axis).** The energy differences due to base stacking and base pairing, which reflect changes in the local arrangement of CpG/^Me^CpG tetramers, were computed in analogy to the thermodynamic cycle used for the free energy calculations: the differences between methylated and un-methylated nucleosome binding energy changes. The energetic components that were included in the stacking and base pairing contributions are detailed in SI material, under the trajectory analysis section. In this plot, the black dots correspond to single mutations 1 to 10 (enumerated in table S1) and the red dots belong to multiple mutations (majGroove, Mixed1 and minGroove, in order of decreasing stability). The associated statistical error is much larger for the enthalpic component due to the typically large interaction energy fluctuations. Although the correlation is only quantitative, this data indicates that the methylation induced nucleosome destabilization is connected to the inability of methylated CpG steps to fit in the required bent conformation of the nucleosome, since it leads to less favorable conformations in term of potential energy interactions.


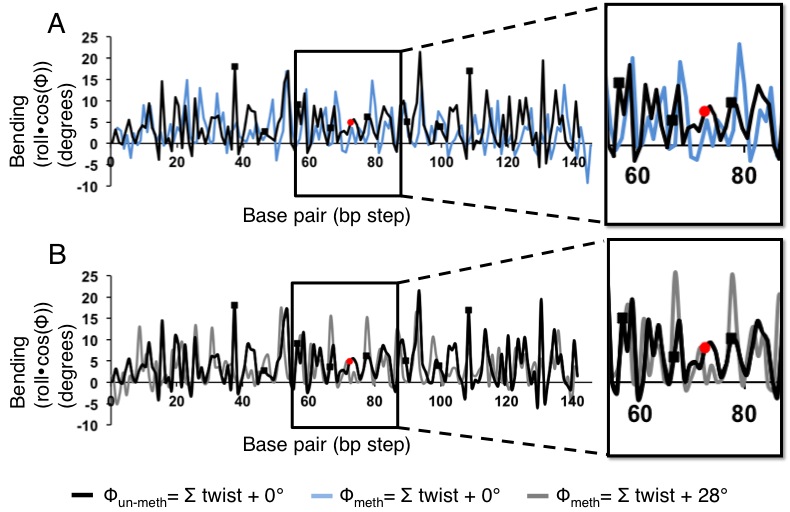


**Supplementary Figure S7.** **Nucleosome-bound methylated and un-methylated DNA bending.** Nucleosome-bound DNA bending of each individual base step when CpG steps are located at positions where the minor groove faces the histone at minor groove positions (black squares) before A) and after methylation B). As explained in the SI material, the roll multiplied by the cosine of the helical phase (Φ) is the major curvature contribution for the bending of the DNA around the histones. In panel A we represent the bending of the DNA un-methylated (black) and methylated (light blue) with the nucleosome dyad positioned 74.5 bps away from the first base pair in the nucleosome (red dot). In these cases the helical phase is identical and has been calculated as the cumulative twist angle along the DNA. In panel B we show the bending of un-methylated (black) DNA, with the nucleosome dyad also positioned at base pair 74.5, and methylated (grey) with the nucleosome dyad displaced to get the best match between un-methylated and methylated bending profiles. In order to minimize the shift of the peaks, the phase angle of the methylated bending has to be increased of 28 degrees. Increase in the phase angle of 28 degrees reflects in moving the dyad by almost 1 base pair step. This data therefore supports that CpG methylation changes the rotational setting by shifting the phase angle and so displacing the DNA with a small movement (as seen in Suppl. Figure S2) as can be detected in the surroundings of the dyad (zoomed panels on the right).
